# Supplementary material for: Blocking IL-10 signaling with soluble IL-10 receptor restores in vitro specific lymphoproliferative response in dogs with leishmaniasis caused by Leishmania infantum
Source: PLoS One. 2021 Jan 19;16(1):e0239171. doi: 10.1371/journal.pone.0239171 (PMC7815104; doi:10.1371/journal.pone.0239171)
Supplement: S1 Raw images — (PDF) [file pone.0239171.s001.pdf]

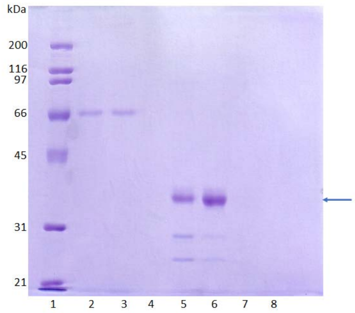

Fig. S1 Evaluation of purified rcasIL-10R1 by SDS–PAGE. RcasIL-10R1 was produced in High-five cells infected with the AcBacΔcc-GP64-casIL-10R1-6H baculovirus construct at MOI 5 for 72 h. Then, rcasIL-10R1 was purified from cell-free and virus-free culture supernatant (SN) by Ni-Sepharose affinity chromatography column. Samples were evaluated by SDS–PAGE using a 15% separating gel: molecular weight markers (lane 1), cell culture SN applied to the chromatographic column (lane 2), flow through (lane 3), and chromatographic elution fractions 1, 2, 3, 4, and 5 (lanes 4, 5, 5, 7, 8, respectively). Arrow indicates a band around 42 kDa corresponding to rcasIL-10R1-6H. This figure was prepared by scanning the gel on a Epson multifunction L375 series printer.

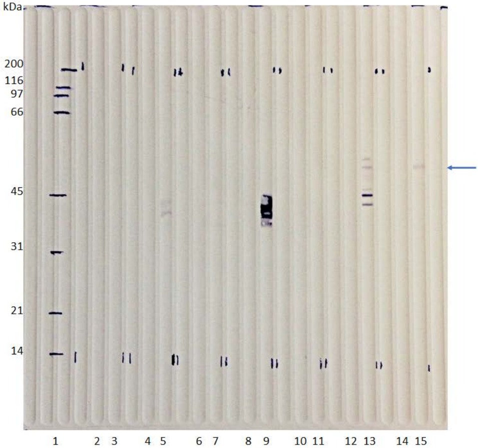

Fig. S2 Fig. S2 Evaluation of purified rcasIL-10R1 by Western blot. RcasIL-10R1 was produced in High-five cells infected with the AcBacΔcc-GP64-casIL-10R1-6H baculovirus construct at MOI 5 for 72 h. Then, rcasIL-10R1 was purified from cell-free and virus-free culture supernatant (SN) by Ni-Sepharose affinity chromatography column. Samples were separated on a 15 % gel as following: molecular weight markers (lane 1), SN from negative control culture (from High five cells infected with AcBacΔcc baculovirus without insert) (lanes 2 and 3), SN from High five cells infected with a baculovirus encoding canine IL-7 and treated without (lanes 4 and 5) or with tunicamycin (lanes 6 and 7), purified canine IL-7 (lanes 8 and 9), SN from negative control culture(from High five cells infected with AcBacΔcc baculovirus without insert) (lanes 10 and 11), SN from High five cells infected with a baculovirus AcBacΔcc-GP64-casIL-10R1-6H (lanes 12 and 13) or purified rcasIL-10R1-6H (lanes 14 and 15). After transferring proteins and blocking the unspecific binding sites, nitrocellulose membrane was incubated with diluent (Tris buffered saline with 0,05 % Tween 20 and 5% skimmed milk) (lanes 2, 4, 6, 8, 10, 12, and 14) or anti-His (C-term, mouse monoclonal antibody) (lanes 3, 5, 7, 9, 11, 13, and 15). Then, the membrane with incubated with anti-mouse Ig alkaline phosphatase-conjugated antibodies. Finally, the membrane was developed with BCIP and NBT. Arrow indicates a band around 42 kDa corresponding to rcasIL-10R1-6H. This figure was prepared by scanning the nitrocellulose membrane on a Epson multifunction L375 series printer. Lanes 2 to 9 are not relevant to the current paper.
